# Production of Chondroitin Sulphate from Head, Skeleton and Fins of *Scyliorhinus canicula* By-Products by Combination of Enzymatic, Chemical Precipitation and Ultrafiltration Methodologies

**DOI:** 10.3390/md13063287

**Published:** 2015-05-27

**Authors:** María Blanco, Javier Fraguas, Carmen G. Sotelo, Ricardo I. Pérez-Martín, José Antonio Vázquez

**Affiliations:** Marine Research Institute (IIM-CSIC), Eduardo Cabello, 6. Vigo, Galicia 36208, Spain; E-Mails: xavi@iim.csic.es (J.F.); carmen@iim.csic.es (C.G.S.); ricardo@iim.csic.es (R.I.P.-M.); jvazquez@iim.csic.es (J.A.V.)

**Keywords:** chondroitin sulphate production, cartilage *S. canicula* wastes, by-products upgrade, process optimization, response surface methodology

## Abstract

This study illustrates the optimisation of the experimental conditions of three sequential steps for chondroitin sulphate (CS) recovery from three cartilaginous materials of *Scyliorhinus canicula* by-products. Optimum conditions of temperature and pH were first obtained for alcalase proteolysis of head cartilage (58 °C/pH 8.5/0.1% (v/w)/10 h of hydrolysis). Then, similar optimal conditions were observed for skeletons and fin materials. Enzymatic hydrolysates were subsequently treated with a combination of alkaline hydroalcoholic saline solutions in order to improve the protein hydrolysis and the selective precipitation of CS. Ranges of 0.53–0.64 M (NaOH) and 1.14–1.20 volumes (EtOH) were the levels for optimal chemical treatment depending on the cartilage origin. Finally, selective purification and concentration of CS and protein elimination of samples obtained from chemical treatment, was assessed by a combination of ultrafiltration and diafiltration (UF-DF) techniques at 30 kDa.

## 1. Introduction

Seafood discards and by-products including whole dead individuals, skins, heads, viscera, bones, cartilage, *etc.* serve as a source for obtaining high value-added products with uses in biomedicine, nutraceutics, feed and cosmetics. In terms of availability of potential raw material for valorization purposes, *Scyliorhinus canicula* might be considered as an alternative source for obtaining valuable compounds. In this regard, *S. canicula* is one of the most discarded species in Northeast Atlantic fisheries. Previously reported data on this species, showed that the percentage of discards might reach 90%–100% in some fisheries [1,2]. In 2012 discards of the Bottom otter trawl (OTB) fleet, operating in the Bay of Biscay and Iberian Waters (ICES Division VIII) accounted for up to 900 t [3]. Besides the importance *of S. canicula* discards as a raw material for obtaining value-added compounds, there is also another fundamental factor contributing to the generation of large quantities of by-products: the onshore fish processing industry. As an example, sales of fresh *S. canicula* in one of the most important fishing ports of Europe, located in Vigo (North-West Spain), accounted for up to 60,700 in for 2013, with an average price of €1.2 per kg (data from http://www.pescadegalicia.com). From these, about 35%–75% of the total weight corresponds to by-products (heads, skin, cartilage, viscera, *etc.*) [4,5,6]. Although much of this waste is already being used, either for fish meal or oil production, it is considered that this kind of utilization produces very little added-value and that, with present technological development, a more valuable and profitable use is possible [7].

Cartilage for biomedical purposes was initially obtained from mammalian sources [8], however since the bovine spongiform encephalopathy outbreak, some concerns arose about the use of by-products from cattle, and more attention has been paid to the use of alternative sources, such as marine organisms for the production of added-value products. The preference for cartilage obtained from marine sources is also explained because previous studies found higher contents of cartilage in sharks in comparison to mammalian sources. Lee and Langer [9] have shown that cartilage in chondrichthyes represents 6%–8% of the total body weight, while mammalian cartilage represents scarcely 0.6%. Chondrichthyes such as *S. canicula* are characterised by a cartilage skeleton mainly composed of the polysaccharide chondroitin sulphate (CS). Chondroitin sulphate is a linear polysaccharide, characterized by a repeating disaccharide unit composed of glucuronic acid (GlcA) and *N*-acetylated galactosamine (GalNAc) sulphated in the carbon 4 (CS-A), 6 (CS-C), both 4 and 6 (CS-E) as well as positions 6 of GalNAc and 2 of GlcA (CS-D) [10]. The CS composition of *S. canicula* has been previously reported to be CS-A, CS-C, CS-D and CS unsulphated [11], whereas in other elasmobranchs such as skates, the composition of CS is different [12]. CS is covalently linked, together with other glycosaminoglycans (keratin sulphate: KS) to an axial protein creating the proteoglycan molecule. Proteoglycans are associated to a collagen matrix constituting the basis of the cartilage tissue. Chondroitin sulphate offers a wide range of applications in medicine such as antioxidant agent, ostheoarthritis treatments, connective tissue repair or anti-tumor drugs [9,13,14,15]. Recently, the combination of CS with other biopolymers such as collagen or hyaluronic acid has attracted much attention in the engineering of biological tissues [16,17,18].

One important aspect regarding the extraction of valuable compounds such as glycosaminoglycans from marine waste materials, is the selection of appropriate processes and the corresponding recovery conditions. Purification processes are commonly optimized using one-factor-at-a-time approaches. However, it is well-known that optimal conditions or interactions between variables cannot be predicted with this methodology. Both problems can be overcome by employing response surface methodology (RSM), a tool used by many researchers to maximize or minimize various independent variables and predict optimal experimental conditions [19,20].

The present work aims to optimize the extraction and purification of chondroitin sulphate from *S. canicula* cartilage wastes, using a set of environmental friendly processes. Firstly, the influence of pH and temperature (*T*) on cartilage hydrolysis with alcalase was studied, and optimized conditions were achieved. Secondly, the optimal concentration of NaOH and ethanol (EtOH) volume for alkaline proteolysis and selective precipitation of CS were obtained. Finally, ultrafiltration process and subsequent diafiltration were developed in order to achieve a high CS purity.

## 2. Results and Discussion

The average (±SD) chemical composition of cartilages from *S. canicula*, expressed as percentage of dry weight, was 52.47 ± 0.10, 55.17 ± 0.74, and 45.19 ± 0.14 of protein for heads, fins and skeletons respectively; 37.66 ± 1.19, 38.70 ± 0.62 and 51.28 ± 0.24 of ash for heads, fins and skeletons respectively; 1.50 ± 0.19, 0.45 ± 0.08 and 0.04 ± 0.01 of fat for heads, fins and skeletons respectively. By difference, the percentage of total carbohydrates was: 8.37 (heads), 5.68 (fins) and 3.45 (skeletons). The content of moisture (as percentage of total weight) was 78.09 ± 0.17, 76.06 ± 1.57 and 70.17 ± 0.25 for heads, fins and skeletons respectively. Similar moisture and fat content, and lower ash and protein content, has been previously described for fin shark cartilage [21].

### 2.1. Enzymatic Hydrolysis of Head Cartilages. Effect of pH and Temperature (T)

**Figure 1:**
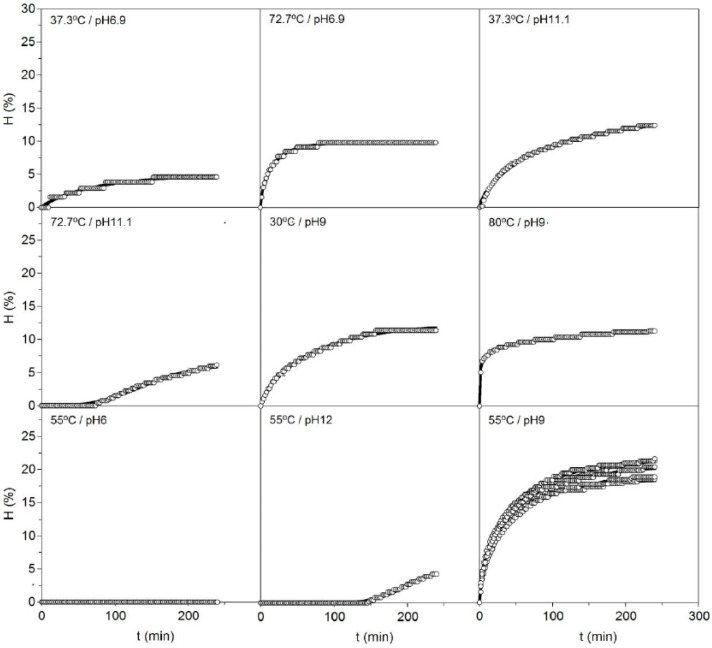
Kinetics of cartilage hydrolysis from *Scyliorhinus canicula* heads using alcalase in each one of the experimental conditions defined in Table 1. The experimental data (symbols) were fitted to the Weibull Equation (4) (continuous line).

Alcalase hydrolysis of head cartilages from *S. canicula* using different conditions of pH and temperature (*T*) clearly showed non-linear patterns with various types of hyperbolic and sigmoid profiles (Figure 1). In this context, the Weibull Equation (4) is a well-known mathematical tool for simulating sigmoid and hyperbolic profiles as well as mixture of both curves [22]. It is also formulated with parameters of clear geometrical meaning and is routinely applied in the modelling of several systems and kinetics in toxicology, food technology and biotechnology [23].

The present experimental data were perfectly described, in all cases, by the equation proposed, obtaining determination coefficients of not less than 0.982. The values of the kinetic parameters and the statistical analysis performed on the numerical fittings are summarized in Table 1. All the parameters were statistically significant (α = 0.05) and autocorrelation was not observed in the residuals distribution (data not shown). For the case (pH 6 and *T* = 55 °C), the values of parameters used as dependent variables (responses) in the subsequent surface response approach and calculation were established as zero.

**Table 1:** Parametric estimations corresponding to the Weibull Equation (4) applied to the enzymatic hydrolysis kinetics at the experimental conditions studied. Independent variables are expressed in natural values in brackets. Numerical values of the parameters are shown with their confidence intervals. Determination coefficients (*R*^2^) and *p*-values from *F*-Fisher test are also summarized. *H*_m_ is the maximum degree of hydrolysis; β is a parameter related with the maximum slope of cartilage hydrolysis; *τ* is the time required to achieve the semi-maximum degree of hydrolysis and *v*_m_ is the maximum hydrolysis rate at the τ-time.

Experimental Conditions	*H*_m_ (%)	*v*_m_ (%·min^−1^)	*τ* (min)	β	*R*^2^	*p*-value
T:−1 (37.3 °C)/pH:−1 (6.9)	5.05 ± 0.31	0.030 ± 0.004	51.51 ± 6.00	0.89 ± 0.10	0.982	<0.001
T:1 (72.7 °C)/pH:−1 (6.9)	9.85 ± 0.04	0.262 ± 0.007	9.82 ± 0.34	0.75 ± 0.03	0.993	<0.001
T:−1 (37.3 °C)/pH:1 (11.1)	14.04 ± 0.46	0.067 ± 0.005	54.65 ± 3.73	0.75 ± 0.03	0.996	<0.001
T:1 (72.7 °C)/pH:1 (11.1)	5.93 ± 0.21	0.045 ± 0.002	139.0 ± 3.17	3.03 ± 0.19	0.991	<0.001
T:−1.41 (30.0 °C)/pH:0 (9.0)	12.80 ± 0.33	0.079 ± 0.005	44.88 ± 2.32	0.80 ± 0.03	0.994	<0.001
T:1.41 (80.0 °C)/pH:0 (9.0)	15.81 ± 2.03	0.082 ± 0.071	14.11 ± 12.42	0.21 ± 0.02	0.992	<0.001
T:0 (55.0 °C)/pH:−1.41 (6.0)	-	-	-	-	-	-
T:0 (55.0 °C)/pH:1.41 (12.0)	4.34 ± 0.15	0.059 ± 0.003	190.23 ± 1.83	7.47 ± 0.44	0.993	<0.001
T:0 (55.0 °C)/pH:0 (9.0)	18.83 ± 0.14	0.225 ± 0.006	19.65 ± 0.45	0.68 ± 0.02	0.997	<0.001
T:0 (55.0 °C)/pH:0 (9.0)	23.44 ± 0.28	0.162 ± 0.006	30.10 ± 0.93	0.60 ± 0.01	0.999	<0.001
T:0 (55.0 °C)/pH:0 (9.0)	19.86 ± 0.16	0.179 ± 0.004	26.70 ± 0.52	0.69 ± 0.01	0.998	<0.001
T:0 (55.0 °C)/pH:0 (9.0)	22.67 ± 0.20	0.209 ± 0.006	23.80 ± 0.55	0.63 ± 0.01	0.998	<0.001
T:0 (55.0 °C)/pH:0 (9.0)	21.06 ± 0.18	0.206 ± 0.006	23.21 ± 0.53	0.66 ± 0.02	0.998	<0.001

**Figure 2:**
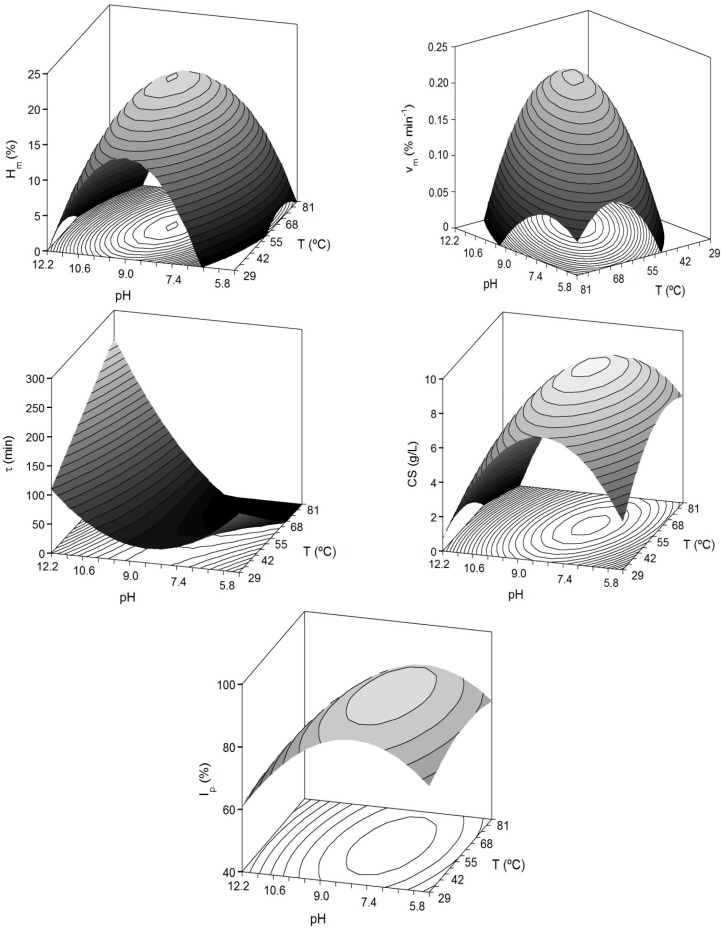
Predicted response surfaces by empirical equations summarized in Table 3 corresponding to the combined effect of pH and *T* on the different dependent variables evaluated for the study of head-cartilages proteolysis by alcalase.

The combined effect of pH and *T* on the kinetic parameters from Equation (4) was studied by means of surface response methodology (Figure 2). Two more dependent variables were also assessed: (1) the concentration of CS was obtained from each sample of hydrolysed cartilage and processed in suboptimal conditions of 0.2 M NaOH and 1 v/v EtOH, according to Murado *et al.* [12]; (2) the index of CS purity in relation to total proteins (*I*_p_ as %). The design and numerical responses of the 2-factor rotatable design are listed in Table 2. For these two responses, the average and corresponding errors (calculated as the intervals of confidence in the five replicated conditions) were: 9.01 ± 0.36 g/L of CS and 89.61% ± 0.53% for *I*_p_.

**Table 2:** Summary of the independent variables (*T*, pH) in the response surface design with the corresponding experimental (*Y*_e_) and predicted (*Y*_p_) results of alcalase head-cartilage hydrolysis, CS production and CS purity regarding total protein (*I*_p_). Natural values of experimental conditions are in brackets.* Determination of CS and *I*_p_ was only done at the end of the hydrolysis time (4 h).

Independent Variables		*H*_m_ (%)		*v*_m_ (% min^−1^)		*τ* (min)		CS (g/L) *		*I*_p_ (%) *	
X_1_: T	X_2_: pH	*Y*_e_	*Y*_p_	*Y*_e_	*Y*_p_	*Y*_e_	*Y*_p_	*Y*_e_	*Y*_p_	*Y*_e_	*Y*_p_
−1 (37.3)	−1 (6.9)	5.05	5.21	0.030	−0.018	51.5	41.7	7.09	6.86	85.12	84.64
1 (72.7)	−1 (6.9)	9.85	11.67	0.282	0.178	9.8	−21.3	9.21	8.35	89.43	86.33
−1 (37.3)	1 (11.1)	14.04	11.67	0.067	0.119	54.7	79.1	3.85	4.74	76.48	74.85
1 (72.7)	1 (11.1)	5.93	5.21	0.045	0.041	139.0	142.1	3.00	3.25	77.42	73.17
−1.41 (30)	0 (9.0)	12.80	14.58	0.079	0.065	44.9	24.7	7.45	7.40	85.25	86.01
1.41 (80)	0 (9.0)	15.81	14.58	0.082	0.148	14.1	24.7	7.38	7.40	82.02	86.01
0 (55)	−1.41 (6.0)	0.00	2.45	0.00	0.055	0.00	24.9	6.00	6.78	80.12	81.69
0 (55)	1.41 (12.0)	4.34	2.45	0.059	0.055	190.2	166.5	2.50	1.69	62.32	65.51
0 (55)	0 (9.0)	18.83	21.17	0.225	0.196	19.7	24.7	9.02	9.01	89.77	89.60
0 (55)	0 (9.0)	23.44	21.17	0.162	0.196	30.1	24.7	9.00	9.01	89.64	89.60
0 (55)	0 (9.0)	19.86	21.17	0.179	0.196	26.7	24.7	9.60	9.01	90.28	89.60
0 (55)	0 (9.0)	22.67	21.17	0.209	0.196	23.8	24.7	8.99	9.01	89.71	89.60
0 (55)	0 (9.0)	21.06	21.17	0.206	0.196	23.2	24.7	8.45	9.01	88.63	89.60

The polynomial models describing the correlation between the variables and response followed the general form defined by Equation (5) and is shown in Table 3.

**Table 3:** Second order equations describing the effect of *T* and pH on alcalase cartilage hydrolysis, CS production and *I*_p_-index (coded values according to criteria defined in Table 1). The coefficient of adjusted determination ($R_{adj}^{\text{2}}$) and *F*-values (*F*_1_, *F*_2_, and *F*_3_) is also shown. S: Significant; NS: Non-significant.

Parameters	*H*_m_	*v*_m_	*τ*	CS	*I*_p_
***b*_0_ (intercept)**	21.17	0.196	24.69	9.01	89.60
***b*_1_ (T)**	-	0.029	-	-	-
***b*_2_ (pH)**	-	-	50.21	−1.80	−5.74
***b*_12_ (TxpH)**	−3.23	−0.069	31.50	−0.74	−0.84
***b*_11_ (T^2^)**	−3.31	−0.045	-	−0.81	−1.80
***b*_22_ (pH^2^)**	−9.42	−0.071	35.73	−2.40	−8.05
$R_{adj}^{\text{2}}$	0.929	0.752	0.874	0.927	0.882
***F*_1_**	53.62 $[F_{9}^{3}=3.86]\Rightarrow S$	5.33 $[F_{8}^{4}=3.84]\Rightarrow S$	28.86 $[F_{9}^{3}=3.86]\Rightarrow S$	39.01 $[F_{8}^{4}=3.84]\Rightarrow S$	23.40 $[F_{8}^{4}=3.84]\Rightarrow S$
***F*_2_**	0.39 $[F_{3}^{8}=8.85]\Rightarrow S$	0.67 $[F_{4}^{8}=6.04]\Rightarrow S$	0.41 $[F_{3}^{8}=8.85]\Rightarrow S$	0.52 $[F_{4}^{8}=6.04]\Rightarrow S$	0.54 $[F_{4}^{8}=6.04]\Rightarrow S$
***F*_3_**	1.17 $[F_{4}^{9}=6.00]\Rightarrow S$	5.09 $[F_{4}^{8}=6.04]\Rightarrow S$	24.76 $[F_{4}^{9}=6.00]\Rightarrow NS$	2.71 $[F_{4}^{8}=6.04]\Rightarrow NS$	21.11 $[F_{4}^{8}=6.04]\Rightarrow NS$

A high proportion of variability (93% for *H*_m_ and CS) was successfully described by the second order equations. In any case, the agreement among experimental and predicted data was always greater than 75% and the robustness was good in all cases; it demonstrated the predictive capacity of the empirical equations in the range of *T* and pH here studied. The results of the multivariate analysis showed significant quadratic negative terms for pH and *T* (*p* < 0.05). This translates graphically in a dome (convex surface) with clear maximum points for the experimental domains of pH and *T* (Figure 2). The inverse response obtained for τ-parameter (concave surface) is in agreement with the fact that when the enzymatic hydrolysis is greater and faster (high *H*_m_ and *v*_m_), the values of τ are shorter.

From the equations summarized in Table 3, the optima values of pH and *T* (pH_opt_ and *T*_opt_) that maximize the corresponding measured responses (*Y*_max_) can be obtained by mathematical optimization using numerical or manual derivation [19] (Table 4). The optimal ranges depending on the variable of response were 55–62.6 °C and 8.14–9 for *T* and pH, respectively. Because all responses are equally important, it has been established the average of the values from Table 4 as the compromise option to select the best condition of pH_opt_ and *T*_opt_. Thus, the values for the subsequent treatment in the alkaline hydroalcoholic solution were: pH = 8.5 and *T* = 58.1 °C.

**Table 4:** Optima values of the two independent variables (*T*_opt_ and pH_opt_) to obtain the maximum responses from the equations defined in Table 3 and for the different dependent variables studied. ^a^ In this case, the optima values of *T* and pH are those that minimize the response of τ.

	*H*_m_	*v*_m_	*τ*	CS	*I*_p_
*T*_opt_ (°C)	55.0	62.6	-	58.3	56.5
pH_opt_	9.0	8.6	9.0 ^a^	8.14	8.23
*Y*_max_	21.17	0.204	-	9.38	90.6

In recent years, alcalase has shown excellent results in the hydrolysis of several fishing wastes, as for instance: Atlantic cod and cattle viscera [24,25], yellowfin tuna heads [26], salmon by-products [27] or cephalopods and shrimp wastewaters [28,29]. Kim *et al.* [30] performed a two-stage enzymatic hydrolysis for CS production from *Isurus oxyrinchus* using a combination of alcalase and flavourzyme. Other proteases have also been evaluated for cartilage hydrolysis in the purification of glycosaminoglycans. Lypaine was applied to the degradation of skate cartilage [31], papain was widely employed in the digestion of different tissues of several origins [11,21,32] and procolax obtained from ray pancreas and commercial papain were compared working on ray cartilage [12]. However, the high hydrolytic capacity, effectiveness on many different substrates and low cost, make alcalase a key enzyme for the recovery and pre-purification of CS from chondrichthyans discards and their by-products.

### 2.2. Enzymatic Hydrolysis of Skeletons and Fins Cartilages

**Figure 3:**
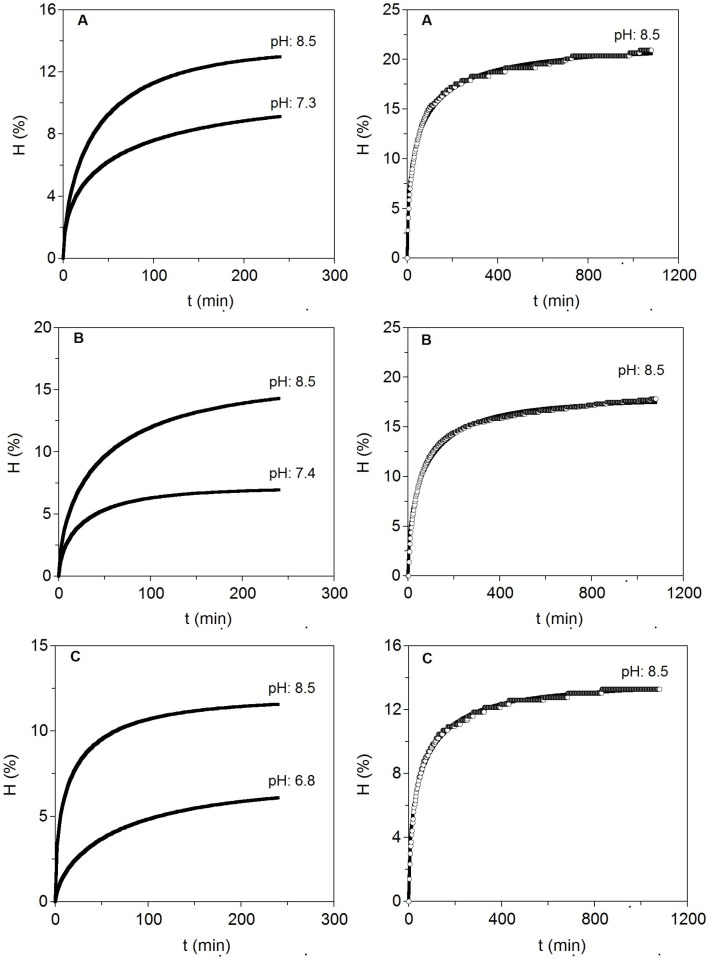
Enzymatic hydrolysis at two pH levels for different cartilages from *S. canicula* wastes (left). To the right, long hydrolysis at the best pH selected are additionally shown. Experimental data were fitted to the Weibull Equation (4). (**A**) Fins; (**B**) Heads and (**C**) Skeletons.

In order to check whether the conditions described for heads were also suitable for the alcalase hydrolysis of other cartilages of *S. canicula* (skeletons and fins), two conditions of pH’s (the initial obtained from the homogeneized cartilages and pH 8.5) at one temperature (58 °C) were assessed. Those modelled kinetics by Equation (4) are displayed in Figure 3 and estimations of the parameters are listed in Table 5.

The results indicated that pH close to 8.5 was better than neutral pH for alcalase hydrolysis of cartilages. It suggests that the optimal conditions for heads can be also extrapolated to hydrolyse cartilages of skeleton and fins with similar positive results. In this context, higher maximum hydrolysis, maximum hydrolysis rate, CS production and CS purity index were significantly generated at alkaline pH. Moreover, the greatest hydrolysis (15.64%) and CS recovery (9.44 g/L) were produced in cartilaginous material from heads as substrate. Different extraction methods, including the use of high intense pulse fields (PEF), or a solvent-free mechanochemical extraction, have been tested for the production of CS from fish cartilage, reporting however, lower contents of CS, 6.92 g/L [33] and 9.33 g/L [34], than those obtained in this study for head shark cartilage. Longer kinetics of hydrolysis (18 h) at 58 °C and pH 8.5 were performed to establish more adequate time needed for enzyme catalysis. More than 8–10 h of proteolysis did not lead to significant increases in the degree of cartilages hydrolysis (less than 10% of variation).

**Table 5:** Parametric estimations corresponding to the Weibull Equation (4) applied to the enzymatic hydrolysis kinetics at the two pH indicated. Numerical values of the parameters are shown with their confidence intervals. In addition, CS concentrations and *I*_p_-index obtained by selective precipitation under standard conditions are also summarized. ^a^ In this case, the kinetics were prolonged up to 18 h.

FINS	*H*_m_ (%)	*v*_m_ (%·min^−1^)	*τ* (min)	β	*R*^2^	CS (g/L)	*I*_p_ (%)
pH: 7.3	10.73 ± 0.06	0.058 ± 0.001	31.82 ± 0.56	0.50 ± 0.00	0.991	5.65	77.5
pH: 8.5	13.59 ± 0.10	0.132 ± 0.003	22.84 ± 0.45	0.64 ± 0.01	0.999	6.50	83.7
^a^ pH: 8.5	21.13 ± 0.10	0.110 ± 0.002	30.82 ± 0.65	0.46 ± 0.01	0.992	6.75	88.3
**HEADS**							
pH: 7.4	7.08 ± 0.01	0.094 ± 0.001	16.53 ± 0.09	0.64 ± 0.00	0.990	7.79	79.9
pH: 8.5	15.64 ± 0.02	0.111 ± 0.001	29.26 ± 0.10	0.60 ± 0.00	0.999	9.44	86.9
^a^ pH: 8.5	17.72 ± 0.07	0.080 ± 0.001	42.60 ± 0.84	0.56 ± 0.01	0.992	9.68	89.6
**SKELETONS**							
pH: 6.8	6.85 ± 0.02	0.037 ± 0.001	42.54 ± 0.26	0.67 ± 0.00	0.997	4.79	76.7
pH: 8.5	11.93 ± 0.29	0.222 ± 0.021	9.25 ± 0.01	0.50 ± 0.04	0.969	6.07	80.4
^a^ pH: 8.5	13.49 ± 0.04	0.074 ± 0.001	31.69 ± 0.84	0.50 ± 0.01	0.995	6.91	87.1

### 2.3. Optimisation of Alkaline Hydroalcoholic Treatment of Enzymatic Hydrolysates

Based on the optimised values described in the previous sections, the hydrolysates of cartilages from different origins (heads, skeletons and fins) were prepared under the following conditions: Hydrolysis time (10 h), *T* = 58 °C, pH = 8.5 (using Tris-HCl buffer 0.1 M), alcalase = 0.1% (v/w) (2.4 AU/kg), solid:liquid ratio (1:1), agitation = 200 rpm. The alcalase hydrolysates were centrifuged at 6000 rpm/20 min and the supernatants were employed in the subsequent treatment with alkaline hydroalcoholic solutions, as described here in the Experimental Section.

CS and *I*_p_ responses (experimental and predicted) from such treatments of *S. canicula* hydrolysates are summarized in Table 6.

Data from CS production and purities were converted into second-order polynomial equations as a function of two independent variables (E and N). The equations describing those effects and their statistical results are represented in Table 7.

**Figure 4:**
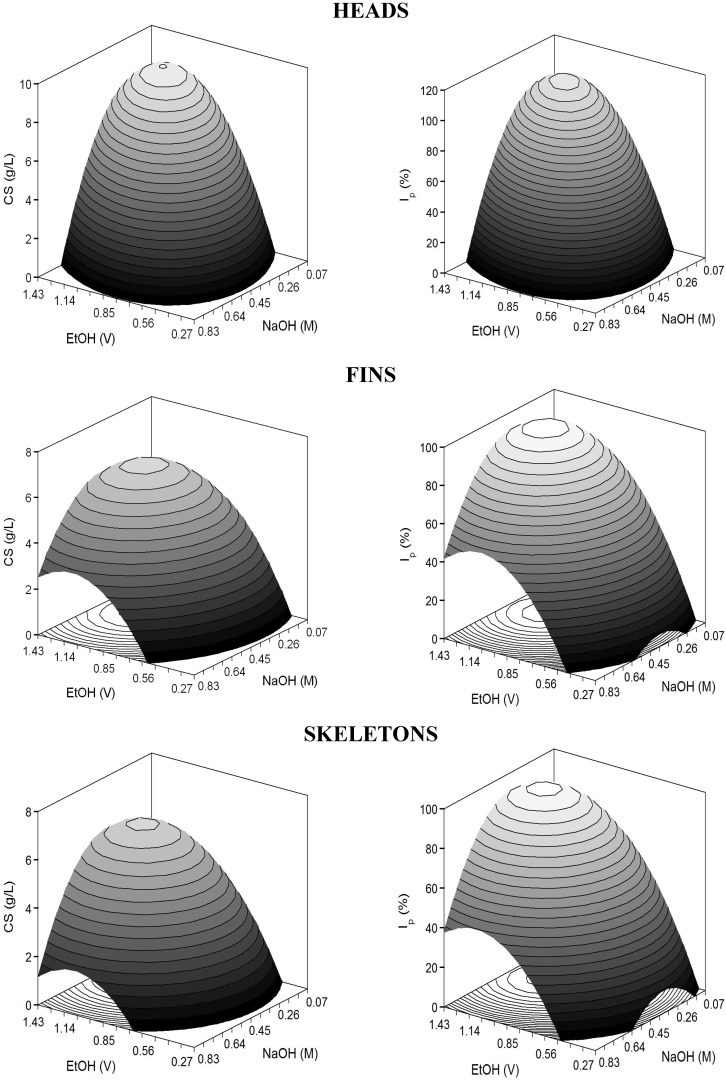
Predicted response surfaces by empirical equations summarized in Table 7 corresponding to the combined effect of NaOH and EtOH on the selective treatment of CS from hydrolysate cartilages of *S. canicula.*

The adjusted coefficients of determination were higher than 0.83 indicating a good correlation between experimental data and theoretical responses. In all cases, responses were significantly affected by positive E and N linear terms and negative quadratic coefficients of both variables (*p* <0.05). The predicted response surfaces were very homogeneous displaying perfect domes (convex surfaces) in the experimental domain executed (Figure 4). Nevertheless, cases of over and under-estimation were observed (Table 6), which do not invalidate the results, and are due to not achieving coefficients of determination nearer to one (Table 7). As described previously, the present $R_{adj}^{\text{2}}$ values revealed good but not perfect agreement among surfaces and experimental data; therefore little lack of fit is commonly obtained.

**Table 6:** Summary of the independent variables (NaOH: N, EtOH: E) in the response surface design with the corresponding experimental (*Y*_e_) and predicted (*Y*_p_) results of selective precipitation of CS from *S. canicula* wastes. Natural values of experimental conditions are in brackets.

		HEADS				FINS				SKELETONS			
Independent Variables		CS (g/L)		*I*_p_ (%)		CS (g/L)		*I*_p_ (%)		CS (g/L)		*I*_p_ (%)	
X_1_: N	X_2_: E	*Y*_e_	*Y*_p_	*Y*_e_	*Y*_p_	*Y*_e_	*Y*_p_	*Y*_e_	*Y*_p_	*Y*_e_	*Y*_p_	*Y*_e_	*Y*_p_
−1 (0.20)	−1 (0.46)	0.25	0.45	4.13	6.63	0.10	0.01	20.56	18.27	0.10	−0.45	22.20	15.48
1 (0.70)	−1 (0.46)	0.50	1.33	5.88	15.01	0.80	1.89	17.14	34.64	0.10	1.36	22.20	37.50
−1 (0.20)	1 (1.24)	0.70	1.75	8.24	19.74	5.74	4.75	86.05	72.17	5.26	4.00	83.63	68.67
1 (0.70)	1 (1.24)	7.32	9.00	87.04	105.17	5.72	5.90	86.34	92.25	5.39	5.81	85.13	90.69
−1.41 (0.10)	0 (0.85)	1.03	0.53	11.54	5.87	1.61	2.40	32.09	44.31	0.10	1.38	22.20	37.49
1.41 (0.80)	0 (0.85)	7.67	6.27	87.08	72.00	5.41	4.53	85.86	70.00	5.14	3.93	83.42	68.54
0 (0.45)	−1.41 (0.30)	0.10	−0.24	2.54	−1.46	0.44	−0.25	22.61	12.57	0.10	−0.40	22.20	16.02
0 (0.45)	1.41 (1.40)	7.62	6.08	88.09	71.34	5.32	5.92	84.78	91.18	5.30	5.88	84.43	91.02
0 (0.45)	0 (0.85)	7.68	7.71	87.44	86.77	5.76	5.67	85.71	85.33	5.26	5.47	84.00	84.19
0 (0.45)	0 (0.85)	7.54	7.71	86.79	86.77	5.46	5.67	85.22	85.33	5.70	5.47	84.71	84.19
0 (0.45)	0 (0.85)	7.70	7.71	86.43	86.77	5.74	5.67	85.91	85.33	5.45	5.47	83.59	84.19
0 (0.45)	0 (0.85)	7.72	7.71	86.19	86.77	5.72	5.67	85.19	85.33	5.47	5.47	84.73	84.19
0 (0.45)	0 (0.85)	7.89	7.71	86.77	86.77	5.66	5.67	84.57	85.33	5.49	5.47	83.93	84.19

**Table 7:** Second order equations describing the effect of N and E on selective precipitation of CS (coded values according to criteria defined in Table 6). The coefficient of adjusted determination ($R_{adj}^{\text{2}}$) and *F*-values (*F*_1_ and *F*_2_) is also shown. S: Significant.

	HEADS		FINS		SKELETONS	
Parameters	CS	*I*_p_	CS	*I*_p_	CS	*I*_p_
***b*_0_ (intercept)**	7.71	86.77	5.67	85.33	5.47	84.19
***b*_1_ (N)**	2.04	23.45	0.76	9.11	0.91	11.01
***b*_2_ (E)**	2.24	25.81	2.19	27.88	2.23	26.59
***b*_12_ (N × E)**	1.59	19.26	−0.18	0.93	NS	NS
***b*_11_ (N^2^)**	−2.17	−24.06	−1.11	−14.17	−1.42	−15.68
***b*_22_ (E^2^)**	−2.41	−26.07	−1.43	−16.83	−1.38	−15.43
$R_{adj}^{\text{2}}$	0.897	0.905	0.885	0.830	0.857	0.849
***F*_1_**	21.97 $\left[F_{7}^{5}=3.97\right]S$	23.88 $\left[F_{7}^{5}=3.97\right]S$	19.54 $\left[F_{7}^{5}=3.97\right]S$	12.71 $\left[F_{7}^{5}=3.97\right]S$	18.92 $\left[F_{8}^{4}=3.84\right]S$	17.85 $\left[F_{8}^{4}=3.84\right]S$
***F*_2_**	0.67 $\left[F_{5}^{8}=4.82\right]S$	0.66 $\left[F_{5}^{8}=4.82\right]S$	0.67 $\left[F_{5}^{8}=4.82\right]S$	0.69 $\left[F_{5}^{8}=4.82\right]S$	0.55 $\left[F_{4}^{8}=6.04\right]S$	0.56 $\left[F_{4}^{8}=6.04\right]S$

The sequential combination of the two-stages for glycosaminoglycan recovery optimised until now led to almost 90% of CS purity against total protein. The best NaOH concentrations and volumes of ethanol for chemical treatment of hydrolysates were (averaging the two responses, CS concentration and purity): 0.64 M and 1.14 volumes for heads, 0.53 M and 1.16 volumes for fins and 0.54 M and 1.20 volumes for skeletons (Table 8). The aforementioned little lack of fit might be also the cause of the over-estimation of *Y*_max_ values showed in Table 8. The optima levels of alkali and alcohol were higher than those found for cartilages of *Raja clavata* [12]. Ethanol has been reported to be an excellent reagent for the selective precipitation of CS, removing the major protein presents in the extract [35]. However, increases in the quantity of ethanol used for the extraction of CS from shark cartilage, did not lead to increases in the yield of the CS obtained [34,36].

**Table 8:** Optima values of the two independent variables (NaOH_opt_ and EtOH_opt_) to obtain the best responses from the equations defined in Table 7 and for the two dependent variables studied (CS concentration and purity).

	HEADS		FINS		SKELETONS	
	CS	*I*_p_	CS	*I*_p_	CS	*I*_p_
NaOH_opt_ (M)	0.63	0.65	0.52	0.54	0.53	0.54
EtOH_opt_ (V)	1.12	1.16	1.14	1.18	1.16	1.24
*Y*_max_	9.24	106.4	6.59	98.6	6.52	97.6

### 2.4. Purification of CS by Ultrafiltration-Diafiltration Processes

**Figure 5:**
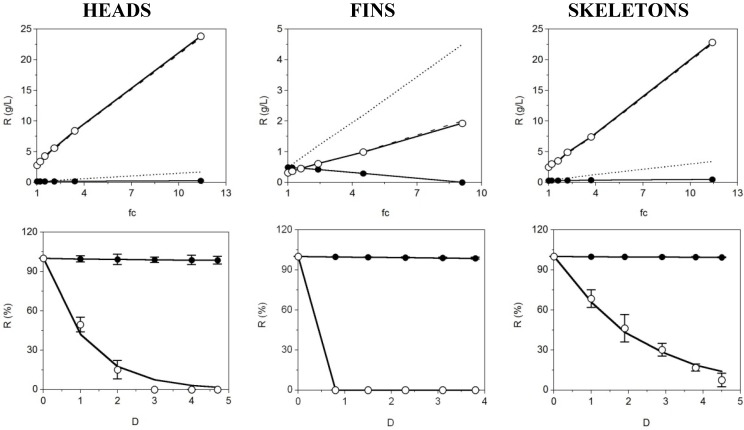
UF-DF process for CS purification from *S. canicula* cartilages of three origins at 30 kDa. Top: Concentration of retained protein (○) and CS (●) in linear relation with the factor of volumetric concentration (fc) showing experimental data (points) and theoretical profiles corresponding to a completely retained solute (discontinuous line). Bottom: Progress of protein (○) and CS (●) retention with the increase of diavolume from DF process (D). Equation (6) was used to fit the experimental data. Error bars are the confidence intervals (α = 0.05; *n* = 2).

The last stage of CS purification was carried out using membrane technologies at a 30 kDa cut-off. Four-liter batches of CS neutralized solutions obtained under the optimal experimental conditions described in previous sections, were purified by a sequence of UF and DF performances. The progress of CS and protein levels *versus* concentration factor by UF is displayed in Figure 5 (Top).

Perfect correlation agreement among theoretical and experimental concentration factor patterns (more than nine-fold in all cases) was reached after the initial 30 kDa UF where the CS concentration from skeletons and heads cartilages was concentrated up to 20–25 g/L. In contrast, the protein was mainly permeated (complete disagreement between predicted and real data) suggesting a lower molecular weight than 30 kDa of the peptidic fraction. The difference of CS recovery comparing origins of the cartilages was due to the lower initial CS content in the fins solutions prepared for UF-DF. The proportion in weight of such cartilage is much lower in comparison to the other fractions, therefore when 4 L of fin solution are obtained, in order to perform representative membrane experiments, the initial concentration of CS is indeed much lower. The filtrate flows during UF processes (concentration step) were maintained in average values of (mL/min): 755, 520 and 900 for fins, head and skeleton samples respectively. The flow falls were inferior to 15% of the average values.

Equation (6) accurately predicted the data of retention dynamics obtained by the DF process (Figure 5, bottom) with high statistical correlation (*R*^2^ > 0.988) (Table 9). All the parameter determinations and the estimation of CS and protein rejection at three diavolumes (*R*_3D_) are also defined in Table 9.

**Table 9:** Parametric estimates from DF purification data (with MWCO of 30 kDa) of CS and proteins fitted to the Equation (6). Determination coefficients (*R*^2^) are also shown. NS: Non-significant.

		CS	Proteins
	*R*_0_	2.52 ± 1.84	100.0 ± 22.6
	*R*_f_	97.4 ± 1.91	0.0
**HEADS**	*s*	0.829 ± 0.189	0.134 (NS)
	*R*^2^	0.996	0.988
	*R*_3D_	1.01	92.6
	*R*_0_	23.2 (NS)	-
	*R*_f_	76.8 ± 41.8	-
**FINS**	*s*	0.985 ± 0.030	-
	*R*^2^	0.999	-
	*R*_3D_	1.02	-
	*R*_0_	20 (NS)	100.0 ± 13.5
	*R*_f_	80 (NS)	0.0
**SKELETONS**	*s*	0.994 ± 0.119	0.561 ± 0.115
	*R*^2^	0.998	0.992
	*R*_3D_	0.36	73.2

The values of the coefficients corresponding to CS, demonstrate that the retention was almost total (*s* ~ 1, *R*_f_ > 76% and *R*_3D_ < 1.1%). In the case of proteins, the permeation of fin solutions was complete at the beginning of the DF and needed more than 3 or 4 relative diavolumes for the heads and skeletons samples, respectively. The complete desalination of retentates was also observed (data not shown). These results reveals the high efficiency of the 30 kDa UF-DF system as a final step to CS retention and recovery and protein discard from *S. canicula* wastes. The purity of CS retentates (in terms of *I*_p_-values) after drying was: 98%, 97% and 96.2% for head, skeleton and fins. If an ulterior purification might be still required, dried samples could return to the alkaline-alcoholic treatment and UF-DF separation, in similar conditions to those described previously. The final yields of CS were (as % of wet weight cartilage): 4.8, 3.3 and 1.5 for heads, fins and skeleton materials, respectively. Membrane separation techniques have been used as the last step of purification of chondroitin sulphate from different cartilage sources, because of the high separation efficiency, different cut-off membranes, ease of scale-up and cost effectiveness [37]. Lignot *et al.* [31] using the UF technique showed lower concentration factors for CS in skate, than those found in this study (up to nine times).

Other methods for the separation/purification of CS are found in literature, including gel filtration [36] or ion-exchange chromatography [38], however the purity of the final CS did not showed any increase in comparison to our results. An initial analysis of chemical composition of sulphate groups indicated that all CS from different types of cartilage were similar with a proportion of sulphation in C6 (6S) and C4 (4S) of 40%–44% and 39%–43% respectively (unpublished data). It also confirmed the validity of the optimisation developed herein. Based on a similar proposal but obtaining different optimal conditions [12], the 6S proportion was 75% in ray cartilage (unpublished data).

## 3. Experimental Section

### 3.1. Cartilage Preparation and Compositional Analysis

Small-spotted catshark (*Scyliorhinus canicula*) individuals obtained approximately 12 h after capture from a local market in Vigo (North-West, Spain) were skinned, heads, skeletons and fins were separated from muscle and processed independently. These materials were heated in a water bath at 80 °C for 30 min to help the manual separation of muscular tissue from cartilage. The cartilages obtained were crushed and homogenized to a particle size of ~1 mm using a grinder and stored at −20 °C until use. The chemical composition of cartilaginous materials was evaluated in triplicate by analysing crude protein, ash, moisture and fat content. Total nitrogen content was determined according to the Kjeldahl method [38] in a DigiPREP HT digestor, DigiPREP 500 fully automatic steam distillation and a TitroLine easy titration unit, and crude protein content was calculated as total nitrogen multiplied by 6.25. Ash was obtained by calcination at 600 °C in a muffle furnace and moisture content determined after heating at 105 °C in an oven until constant weight. Lipid content was determined by the methodology of Bligh and Dyer [39]. Finally, the total carbohydrate content was estimated by the difference between total weight (subtracting protein, fat and ash) and moisture content.

### 3.2. Analytical Determinations

Total soluble proteins (Pr) of CS solutions were determined by the method of Lowry *et al.* [40]; CS, as glucuronic acid, was quantified by the method of Van den Hoogen *et al.* [41], according with the modifications of Murado *et al.* [42]. This modified method is mainly efficient and sensitive for glucuronic acid without sulphation. Thus, keratan sulphate (d-galactose + 6-sulphate-*N*-acetylglucosamine) is not detected and dermatan sulphate (also known as chondroitin sulphate B: Iduronic + 4-sulphate-*N*-acetylglucosamine) as well as heparan sulphate (2-sulphate-glucuronic or iduronic acid + 6-sulphate-*N*-sulphoglucosamine) proved to be less sensitive to that reaction (25% of the glucuronic acid sensitivity). Additionally, previous results [11,36] have indicated almost no presence of heparan sulphate in *S. canicula* and *Sphyrna Lewini* (another similar shark) and more than 80% of CS of the total glycosaminoglycans in the cartilage composition. The presence of hyaluronic acid (equally well determined by m-hydroxydifenyl reaction) in the proteoglycan matrix of fin cartilage from *S. Lewini* has not been demonstrated and its value is lower than 10% of total glycosaminglycans in *S. canicula* cartilage [11,36]. Therefore, quantification of CS as proposed is adequate and does not invalidate the results herein obtained. CS purity index (*I*_p_), defined as *I*_p_ (%) = CS × 100/(CS + Pr), was also calculated in all purification stages.

### 3.3. Enzymatic Hydrolysis of Cartilages

Cartilages were hydrolysed using Alcalase 2.4 L from *Bacillus licheniformis* (Novozyme Nordisk, Bagsvaerd, Denmark). The enzyme/substrate ratio was 2.4 U/kg of fresh cartilage and the solid:liquid ratio was (1:1). Hydrolysis was prepared using a stirred (200 rpm) and thermostatted reactor (100 mL) connected to pH and temperature electrodes and coupled to an auto-titrator (Metrohm). *T* and pH conditions were established according to a full factorial design of second order, as it is described in the Experimental Section. pH levels of each point of the experimental design were adjusted by adding 0.2 M NaOH, and the pH was maintained constant during the hydrolysis reaction by automatic addition of 0.2 M NaOH. After 4 h of hydrolysis, samples were inactivated by boiling (10 min), cooled in an ice-water bath and centrifuged (6000× *g*, 20 min). Sediments were discarded and the supernatants stored at −20 °C until further analysis. The extent of enzymatic hydrolysis was determined by the pH-Stat method [43], which allows the estimation of degree of hydrolysis (*H*) based on amount of alkali needed to maintain the pH at the desired level. Thus, *H* (in %) could be obtained according to the following expression being the percent ratio between the total number of peptide bonds cleaved and the total number of peptide bonds in the original protein:

(1) $$H=\frac{B\;N_{b}}{\alpha \;M_{p}\;h_{tot}}$$

where, *B* is the volume (mL) of 0.2 M NaOH consumed during hydrolysis; *N*_b_ is the normality of NaOH; *M*_p_ is the mass (g) of initial protein (*N* × 6.25); *h*_tot_ is the total number of peptide bonds available for proteolytic hydrolysis (8.6 meq/g), and α is the average degree of dissociation of the amino groups in the protein substrate, and was calculated as follows:

(2) $$\alpha =\frac{10^{pH-pK}}{1+10^{pH-pK}}$$

The p*K* value is dependent on the temperature of hydrolysis (in *K* degrees), therefore it can be also calculated according to the following expression:

(3) $$pK=2400\left(7.8+\frac{298-T}{298\;T}\right)$$

### 3.4. Mathematical Modelling of the Proteolysis Kinetics

The non-linear kinetics of *S. canicula* cartilage hydrolysis mediated by alcalase, under different pH and *T* conditions, were fitted to the Weibull equation [22,44]:

(4) $$H=H_{m}\left\{1-\exp\left[-\ln2{\left(\frac{t}{\tau }\right)}^{\beta }\right]\right\}\;\text{with}\;v_{m}=\frac{H_{m}\;\beta \;\ln2}{2\;\tau }$$

where, *H* is the degree of hydrolysis (%); *t* is the time of hydrolysis (min); *H*_m_ is the maximum degree of hydrolysis (%); β is a parameter related with the maximum slope of cartilage hydrolysis (dimensionless); τ is the time required to achieve the semi-maximum degree of hydrolysis (min) and *v*_m_ is the maximum hydrolysis rate at the τ-time (% min^−1^).

### 3.5. Experimental Designs and Statistical Analysis

Two different experimental designs were performed in the present work. First, the effect of temperature (*T*) and pH on the hydrolysis degree of head cartilages (according to kinetic parameters from Equation (4)) and catalyzed by alcalase, was studied. Then, the concentration of NaOH (N) and the volumes of ethanol (E) needed for the final alkaline proteolysis of proteoglycan and selective precipitation of CS against proteins, was optimized. In both cases, the factorial experiments were rotatable second order designs with five replicates in the centre of the experimental domains [45].

The conditions of the independent variables studied for the enzymatic hydrolysis of shark materials were: *T* in the range 30–80 °C and pH in the range 6–12. The rest of experimental conditions were kept constant (see enzymatic hydrolysis section). The experiments of CS recovery from the enzymatic hydrolysates obtained in the optimal conditions from previous design, were carried out by slow addition and with moderate agitation at room temperature, and hydroalcoholic solutions of NaOH in the required proportions to obtain reaction mixtures with the preestablished values of N and E in the following intervals: N (0.1–0.8 M) and E (0.3–1.4 v). In order to improve the subsequent CS recovery in water, 2.5% NaCl was added to all alkaline hydroalcoholic mixtures. After a period of 2 h in agitation, the suspensions were centrifuged (6000× *g*; 20 min) and the sediments were redissolved with water and neutralized using 6 M HCl. The encoding procedure of the variables was performed by the following formulas:
CodificationDecodification*V*_c_ = (*V*_n_ − *V*_0_)/Δ*V*_n_*V*_n_ = *V*_0_ + (Δ*V*_n_ × Δ*V*_c_)*V*_n_: Natural value of the variable to codify *V*_0_: Natural value in the centre of the domain *V*_c_: Codified value of the variable Δ*V*_n_: Increment of *V*_n_ per unit of *V*_c_

Both expressions of the independent variables, codified and natural values, in each experimental run are summarized in Table 1, Table 2 and Table 6.

Orthogonal least-squares calculation on factorial design data, were used to obtain empirical equations describing the different dependent variables studied (*Y*), each one related to *T* and pH for enzymatic hydrolysis and N and E for CS production. The general form of the polynomial equations is:

(5) $$Y=b_{0}+\sum_{i=1}^{n}b_{i}X_{i}+\sum_{\begin{array}{l} i=1\\ j>i \end{array}}^{n-1}\sum_{j=2}^{n}b_{ij}X_{i}X_{j}+\sum_{i=1}^{n}b_{ii}X_{i}^{2}$$

where *Y* represents the response to be modelled; *b*_0_ is a constant coefficient, *b*_i_ is the coefficient of linear effect, *b*_ij_ is the coefficient of interaction effect, *b*_ii_ the coefficients of squared effect, *n* is the number of variables and *X*_i _ and *X*_j_ define the independent variables. The statistical significance of the coefficients was verified by means of the Student *t*-test (α = 0.05), goodness-of-fit was established as the adjusted determination coefficient ($R_{adj}^{\text{2}}$) and the model consistency by the Fisher *F* test (α = 0.05) using the following mean squares ratios:

the model is acceptable whenF1 = Model/Total error$F1\geq F_{den}^{num}$F2 = (Model + Lack of fitting)/Model$F2\leq F_{den}^{num}$F3 = Total error/Experimental error$F3\leq F_{den}^{num}$

$F_{den}^{num}$ are the theoretical values to α = 0.05 with the corresponding degrees of freedom for numerator (num) and denominator (den). All fitting procedures, coefficient estimates and statistical calculations were performed on a Microsoft Excel spreadsheet.

### 3.6. Ultrafiltration-Diafiltration Process

CS neutralized solutions were subjected to ultrafiltration-diafiltration (UF-DF) using a membrane (Prep/Scale-TFF: Spiral polyethersulfone membrane of 0.56 m^2^, Millipore Corporation, Bedford, MA, USA) of 30 kDa molecular weight cut-off (MWCO). The operation mode was the following: An initial phase of ultrafiltration (UF) at 40 °C with total recirculation of retentate was performed, immediately followed by a diafiltration (DF) step. During UF, the inlet pressure remained constant (<1 bar) to determine the drops of flow rate due to the increased concentration of the retentate and to possible adhesions to the membrane. The final retentate (after DF) was lyophilized and stored at 4 °C for further analysis. Permeate of the UF step was analysed and finally discarded. For modelling the membrane process, we fixed a DF with constant volume (filtration flow = water intake flow), where the concentration of a permeable solute in the retentate was predicted by using the first-order equation [12]:

(6) $$R=R_{f}+R_{0}\exp\left[-\left(1-s\right)D\right]$$

where, *R* is the concentration of permeable protein or CS in the retentate (% from the level at initial DF), *R*_0_ is the permeate concentration (%), *R*_f_ is the asymptotic and retentate concentration (%), *D* is the relative diavolume (volume of added water/constant retentate volume) and *s* is the specific retention of protein or CS with variation between 0 (the solute is filtered as the solvent) and 1 (the solute is totally retained). Thus, using normalized values (%): *R*_0_ + *R*_f_ = 100, with *R*_0_ = 0 if all protein or CS are permeable. In addition, the percentage of protein or CS eliminated by three diavolumes (*R*_3D_) was calculated by substituting in Equation (6) the value of parameter *D* by 3.

### 3.7. Numerical Methods for Non-Linear Curves Modelling

Cartilage hydrolysis and UF-DF data were modelled by minimisation of the sum of quadratic differences between observed and predicted values, using the non-linear least-squares (quasi-Newton) method provided by the macro “Solver” of the Microsoft Excel spreadsheet. Confidence intervals from the parametric estimates (Student’s *t* test) and consistence of mathematical models (Fisher’s *F* test) and residual analysis (Durbin-Watson test) were evaluated by “SolverAid” macro [46].

## 4. Conclusions

A complete optimization of the different processes involved in the CS recovery and purification from cartilage wastes of *S. canicula* have been developed. Two experimental designs, incorporating kinetic approaches, were carried out to define the effect of pH and temperature on alcalase activity and the joint capacity of NaOH and EtOH on CS selective precipitation. Both proposals were successfully solved obtaining optimal conditions as follows: pH = 8.5 and *T* = 58 °C for enzymatic hydrolysis, and 0.53–0.64 M of NaOH and 1.14–1.20 volumes of EtOH for chemical treatment. In addition, we can indicate that the head wastes are the best source of CS production from *S. canicula*. Finally, the extracts from alkaline hydroalcoholic treatment were processed by UF-DF protocols at 30 kDa of MWCO for the differential retention of CS and concomitant rejection of protein material. Both objectives were successfully reached with total concentration and recoverability of CS as well as protein elimination using no more than 3–5 diavolumes in the DF step.

Our results showed that *S. canicula* is a good source of CS and such bioproduction is an excellent alternative for the valorization of discards and its by-products. However, further physicochemical studies are required to characterize completely the type of CS involved and the sulphation pattern presents in the glycosaminoglycan purified. These experiments exceed the objectives reported in the present work.
